# Applicability of contemporary quality indicators in vestibular surgery—do they accurately measure tumor inherent postoperative complications of vestibular schwannomas?

**DOI:** 10.1007/s00701-021-05044-2

**Published:** 2021-12-02

**Authors:** Stephanie Schipmann, Sebastian Lohmann, Bilal Al Barim, Eric Suero Molina, Michael Schwake, Özer Altan Toksöz, Walter Stummer

**Affiliations:** Department of Neurosurgery, University Hospital Münster, Albert-Schweitzer-Campus 1, 48149 Münster, Germany

**Keywords:** Vestibular schwannoma, Quality indicator, Facial nerve palsy, Readmission, Reoperation, Surgical site infection, Acoustic neurinoma

## Abstract

**Background:**

Due to rising costs in health care delivery, reimbursement decisions have progressively been based on quality measures. Such quality indicators have been developed for neurosurgical procedures, collectively. We aimed to evaluate their applicability in patients that underwent surgery for vestibular schwannoma and to identify potential new disease-specific quality indicators.

**Methods:**

One hundred and three patients operated due to vestibular schwannoma were subject to analysis. The primary outcomes of interest were 30-day and 90-day reoperation, readmission, mortality, nosocomial infection and surgical site infection (SSI) rates, postoperative cerebral spinal fluid (CSF) leak, facial, and hearing function. The secondary aim was the identification of prognostic factors for the mentioned primary outcomes.

**Results:**

Thirty-day (90-days) outcomes in terms of reoperation were 10.7% (14.6%), readmission 9.7% (13.6%), mortality 1% (1%), nosocomial infection 5.8%, and SSI 1% (1%). A 30- versus 90-day outcome in terms of CSF leak were 6.8% vs. 10.7%, new facial nerve palsy 16.5% vs. 6.1%. Hearing impairment from serviceable to non-serviceable hearing was 6.8% at both 30- and 90-day outcome. The degree of tumor extension has a significant impact on reoperation (*p* < 0.001), infection (*p* = 0.015), postoperative hemorrhage (*p* < 0.001), and postoperative hearing loss (*p* = 0.026).

**Conclusions:**

Our data demonstrate the importance of entity-specific quality measurements being applied even after 30 days. We identified the occurrence of a CSF leak within 90 days postoperatively, new persistent facial nerve palsy still present 90 days postoperatively, and persisting postoperative hearing impairment to non-serviceable hearing as potential new quality measurement variables for patients undergoing surgery for vestibular schwannoma.

## Introduction

Vestibular schwannomas (VS) are slow growing, primarily benign brain tumors that arise from Schwann cells covering the vestibular portion of the 8th cranial nerve [6, 17].

Continuous advances in surgical procedures have led to considerably improved postoperative mortality and morbidity rates [48]. However, complete tumor resection while preserving facial and hearing functions remains highly challenging [31].

Efforts of mitigating complications in VS surgery have further gained attention considering the increasing relevance of standardized quality measures. Aiming at realizing the highest quality of delivered care at acceptable costs, risk stratification based on standardized quality indicators has become a central issue in many medical fields, including neurosurgery [30, 47]. In light of rising costs of health care delivery and changes in the policy of health care providers, reimbursement decisions have progressively been based on quality measures [26, 27].

While prognostic factors of VS on facial nerve preservation have extensively been discussed, analyses of further quality-related outcome measures are sparse [4, 15, 16, 22, 56]. Presently used quality indicators have often been developed for all neurosurgical procedures and focus mainly on the first 30 postoperative days [45]. Hence, we aimed to analyze their applicability to the disease-inherent characteristics of VS and to evaluate the necessity of longer observation periods. The secondary aim of the study was the identification of associated risk factors of primary outcome measures. Understanding variables that increase the likelihood of adverse events as potential quality indicators will help defining more tumor-specific standards in vestibular surgery.

## Methods

### Patient data

All patients that underwent microsurgical VS resection between January 2013 and May 2019 at the Department of Neurosurgery, University Hospital Münster were retrospectively included in this study. Patients with Hannover T4 tumors operated in 2013 and 2014 were also subject to previous analysis [31]. Patients with neurofibromatosis type II and bilateral VS were excluded from the analysis due to a different treatment approach.

Baseline characteristics such as age, sex, presenting symptoms, surgical details, and patient’s comorbidities were obtained from the electronic medical records of each patient.

Hearing function was stratified into normal hearing (Gardner Robertson scale (GS) grade 1), serviceable hearing (GS grade 2), and non-serviceable hearing (GS ≥ 3) [19].

Facial nerve function was graded according to the House & Brackman classification [25]. Tumor growth was stratified according to the Hannover tumor extension grading scale [41] and size was measured using the largest tumor diameter including the intracanalicular portion.

The extent of resection (EOR) was categorized in complete resection (CR) (postoperative MR without contrast enhancement), near total resection (NTR) (any form of linear enhancement), and subtotal resection (STR) (measurable residue enhancement).

Secondary diagnoses were classified using the 19 items from the Charlson comorbidity index (CCI) and the age-adjusted CCI (ACCI) was calculated [9, 10]. Only comorbidities present on admission were considered part of the patient’s preadmission diagnosis profile.

### Outcomes of interest

All adverse events were merged into the different categories of currently established quality metrics [12, 13, 34, 43, 45–47, 50]. The primary outcomes of interest were 30-day and 90-days reoperation, readmission, mortality, nosocomial infection and surgical site infection (SSI) rates, postoperative cerebral spinal fluid (CSF) leak, hearing, and facial function. Data regarding EOR and recurrence were obtained.

Only unplanned reoperations and readmission were considered. Readmissions within 30/90 days to other hospitals were not recorded. Reasons for readmission were classified into CSF leak, SSI, new facial palsy, and unrelated, e.g., medical reasons. Reasons for reoperation comprised postoperative hemorrhage, SSI, CSF leak, and hydrocephalus.

New persistent facial nerve dysfunction was defined as HB grade III or higher function persisting for more than 3 months postoperatively. The secondary aim of the study was the identification of risk factor for the mentioned outcome measures. The study was approved by the local ethics committee (2018–128-f-S).

### Surgery and intraoperative neuromonitoring, routine follow-up

Prior to surgery, the various treatment options comprising “wait and see,” radiotherapy, or surgical resection were discussed with the patient, depending on tumor size, patient’s wish, and comorbidities.

In case of small tumors (approx. < 2 cm diameter), all patients were seen by both a neurosurgeon and a radio-oncologist to discuss surgical and radiation options.

A retrosigmoid approach was followed in all cases and resection was carried out as described before [31]. Routine monitoring of cranial nerves V (electromyography—EMG), VII (EMG, motor evoked potential—MEP, direct nerve stimulation—DNS), and VIII (acoustic evoked potential—AEP), as well as somatosensory evoked potentials (SSEP), were applied using ISIS Xpert (Inomed, Emmendingen, Germany). Direct stimulation of the tumor surface, monitored by facial EMG, was used to identify the position of the facial nerve prior to tumor resection. In addition, transcortical stimulation for monitoring of the facial nerve motor function was applied. Depending on the tumor size and extension additional cranial nerves were monitored. CR was performed whenever possible. The main aim of surgery was to preserve facial nerve function while resecting as much tumor as securely possible. Patients with STR only were either followed up with serial imaging or scheduled for radiation treatment after surgery. Decision making was based on the individual case.

Patients were routinely seen 6 weeks postoperatively for clinical evaluation and after 3 months for MRI follow-up in our outpatient department. In case of CR, MRI follow-up was performed yearly, and in patients with a residual tumor semiannual. In case of tumor recurrence, the various options (wait-and-see, radiosurgery and surgery) are discussed with the patients in consideration of tumor size, extension, patients wish, hearing, and facial nerve function.

### Statistical analyses

Statistical analyses were performed using IBM SPSS Statistics 26.0 software (IBM, Armonk, New York, USA). We used absolute and relative frequencies for categorical variables and median and interquartile range for continuous variables. Chi-square test was used for categorical variables and *t*-test and Mann–Whitney *U* test for continuous variables, as appropriate. All variables significant in the bivariate analyses were entered into a multivariable logistic regression model. Odds ratios (ORs) were calculated and obtained with corresponding 95% confidence intervals (CIs). Time to event analysis was conducted using log-rank test. A two-sided probability value *p* of less than 0.05 was considered statistically significant.

## Results

### Demographic data

A total of 103 patients were included in this study, of whom 57.3% were female and 42.7% were male. A large majority of cases presented with newly diagnosed tumors (*n* = 90, 87.4%).

Tumor location was distributed almost equally between the left (*n* = 52, 50.5%) and right (*n* = 51**,** 49.5%) site with a median tumor diameter of 2.1 cm (IQR 1.2). The majority of patients presented with a Hannover tumor extension grading scale T4a tumor (*n* = 46, 44.7%).

The median follow-up time was 20 (IQR 35.75) months. Demographics and baseline characteristics of the study population are depicted in Table 1.

**Table 1:** Baseline characteristics of the 103 included cases

		*n*	%
Age	*Median (IQR)*	*53 (18)*	-
Sex	Male	44	42.7
	Female	59	57.3
Urgency of admission	Elective	101	98.1
	Emergency	2	1.9
Primary diagnosis		90	87.4
Recurrent tumor		13	12.6
	Previous tumor resection	7	6.8
	Previous tumor resection	7	6.8
Focal neurological deficit preop		40	38.8
Facial nerve function preop	HB I	94	91.3
	HB II	7	6.8
	HB III	1	1.0
	HB IV	1	1.0
Hearing function preop	Normal	21	20.4
	Serviceable	71	68.9
	Non-serviceable	11	10.7
Tumor site	Left	52	50.5
	Right	51	49.5
Tumor size (cm)	*Median, IQR*	2.1 (1.2)	-
Hannover tumor extension grading scale	T1	2	1.9
	T2	14	13.6
	T3a	13	12.6
	T3b	17	16.5
	T4a	46	44.7
	T4b	11	10.7
Hydrocephalus prior to surgery		9	8.7
ECOG preop	0	91	88.3
	1	10	9.7
	2	2	1.9
ASA preop	1	22	24.4
	2	57	63.3
	3	11	12.2
ACCI admission	*Median (IQR)*	1 (3)	-
Number of secondary diagnoses	*Median (IQR)*	0 (0)	-
Depression		11	10.7
Nicotine abuse		11	10.7
Leukocytosis (> 10.900 per μl) preop		13	12.6
CRP (> 1 mg/dl) preop		5	4.9
Length of surgery (min)	*Median (IQR)*	350 (128)	128
Extent of resection	CR	43	41.7
	NTR STR	20 39	19.6 38.2
Length of stay	*Median (IQR)*	8 (3)	-
Length of stay on ICU	*Median (IQR)*	1 (0)	-

*IQR*, interquartile range; *preop*, preoperative; *ECOG*, Eastern Cooperative Oncology Group; *ASA*, American Society of Anesthesiologists; *ACCI*, age-adjusted Charlson comorbidity index; *CRP*, C-reactive protein; *CR*, complete resection; *STR*, subtotal resection; *ICU*, intensive care unit

### Outcome variables

Outcome rates and the time of onset after surgery are summarized in Table 2.

**Table 2:** Incidence of postoperative adverse events

Outcome	Cumulative incidence *n* (%)	Time to event (days)	
		Median	IQR
30-day reoperation	**11 (10.7)**	**3.0**	**13**
* Postoperative hemorrhage*	6 (5.8)	2.5	3
*CSF leak*	2 (1.9)	17	-
*Surgical site infection*	1 (1.0)	18	-
*Hydrocephalus*	2 (1.9)	1.5	-
90-day reoperation	**15 (14.6)**	**4.0**	**32**
*Postoperative hemorrhage*	6 (5.8)	2.5	3
*CSF leak*	6 (5.8)	44	40
*Surgical site infection*	1 (1.0)	18	-
*Hydrocephalus*	2 (1.9)	1.5	-
30-day readmission	**10 (9.7)**	**7**	**18**
*CSF leak*	5 (4.9)	13.5	18
*Surgical site infection*	1 (1.0)	10	-
*New facial nerve palsy*	3 (2.9)	3	-
Unrelated*	1 (1.0)	23	-
90-day readmission	**14 (13.6)**	**15**	**26**
*CSF leak*	6 (5.8)	15	26
*Surgical site infection*	1 (1.0)	10	-
*New facial nerve palsy*	3 (2.9)	3	-
Unrelated*	4 (3.9)	44.5	51
30-day mortality	**1 (1.0)**	**2**	**-**
90-day mortality	**1 (1.0)**	**2**	**-**
30- and 90-day nosocomial infection	**6 (5.8)**	**15.5**	**14**
*Meningitis*	3 (2.9)	7	-
*Pneumonia*	1 (1.0)	14	-
*Surgical site infection*	1 (1.0)	17	-
*Sepsis*	1 (1.0)	30	-
30-day surgical site infection	1 (1.0)	17	-
90-day surgical site infection	1 (1.0)	17	-
30-day CSF leak	**7 (6.8)**	**5**	**12**
90-day CSF leak	**11 (10.7)**	**14**	**38**
30-day facial nerve dysfunction**	**17 (16.5)**	**N/A**	**-**
90-day persistent facial nerve dysfunction**	**6 (6.1)**	**N/A**	**-**
30-day non-serviceable hearing***	**7 (6.8)**	**N/A**	**-**
90-day non-serviceable hearing***	**7 (6.8)**	**N/A**	**-**

^*^Unrelated = worsening of general condition not related to surgery

^**^Defined as new facial nerve palsy ≥ HB grade III

^***^Worsening of hearing from Gardner-Robinson grade 1/2 to grade ≥ 3 (non-serviceable hearing)

*N/A*, not applicable; *CSF*, cerebrospinal fluid

### Reoperation

Patients were reoperated within 30 days in 10.7% (*n* = 11) of cases. The main reason for early reoperation was a postoperative hemorrhage, which occurred in 6 cases (5.8%) after a median of 2.5 days (IQR 3). Time to event analysis revealed that reoperation due to hydrocephalus and hemorrhage occurred within the first days after surgery and significantly earlier than reoperations due to CSF leak or SSI (*p* = 0.036). Most reoperations occurred during index admission (72.7%, *n* = 8).

In comparison, the 90-day reoperation rate was 14.6%; the difference between the 30-day period was based only on more patients with CSF leak.

### Readmission

The 30-day readmission rate was 9.7% (*n* = 10). Most readmissions were based on a CSF leak (*n* = 5, 4.9%). In three cases (2.9%), a secondary onset of a facial nerve dysfunction let to readmission. These patients were treated with intravenous acyclovir and steroids and in all cases, the facial nerve function normalized after a few weeks. There was no significant difference regarding the timing of readmission and the reason (*p* = 0.075). The 90-day readmission rate was 13.6%. Four more patients were readmitted between days 31 and 90, the reason for readmission was mainly unrelated (75%) and in one case due to CSF leak (25%).

### Mortality rate

The 30- and 90-day mortality rates were 1%. The patient died due to pulmonary embolism.

### Nosocomial infection and surgical site infection

A nosocomial infection was observed in 5.8% of cases (*n* = 6). Three patients (2.9%) developed meningitis after a median of 7 days. The SSI rate in our collective was 1% (*n* = 1). No further infections manifested between days 31 and 90, consequently, there was no difference between the 30-day and 90-day nosocomial and surgical site infections rates.

### Postoperative CSF leak

The 30-day vs. 90-day postoperative CSF leak rate was 6.8% (*n* = 7) vs. 10.7% (*n* = 11). Nine patients (8.7%) required a lumbar drain and surgical revision was required in 6 cases (5.8%). The median time to onset of a CSF leak was 14 days (IQR 38). The maximal time between surgery and CSF leak was 71 days.

### Persistent facial nerve dysfunction

Most patients (*n* = 94, 91.3%) presented with intact facial nerve function prior to surgery (Table 1).

**Fig. 1:**
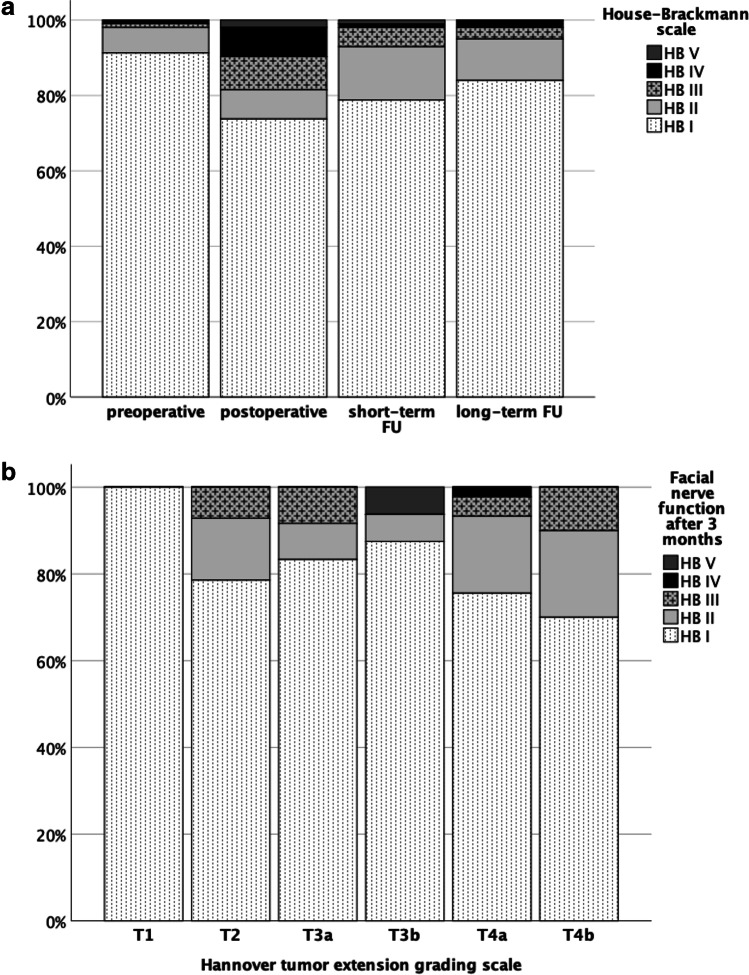
**a** Course of the facial nerve function regarding the different time points (preoperative, postoperative (within 30 days) and short-term FU (90 days) and after > 3 months (long-term FU). FU, follow-up. **b** Facial nerve function three months postoperative in relation to the tumor extension according to the Hannover classification. There is a clear tendency that a higher Hannover grade leads to a higher probability of a persistent postoperative facial nerve dysfunction without reaching statistical significance (*p* = 0.980). HB, House& Brackman

Figure 1a shows the course of the facial nerve function regarding the different time points before and after surgery. One-quarter of patients (*n* = 26, 25.2%) showed an initial worsening of facial function within the first 30 days after surgery. However, a persistent facial nerve dysfunction still present at 90 days postoperatively was observed in only 6 cases (6.1%), indicating that facial nerve functions improves during the early postoperative course. In detail, 4 patients worsened from normal function (HB I) to HB III, and one patient each from HB I to HB V and HB II to HB III. Except for one patient, all patients that showed a new postoperative facial nerve dysfunction improved in long-term follow-up. In half of those cases (50%, *n* = 3), facial function was normalized in long-term follow-up.

Figure 1b shows the outcome regarding facial nerve function 90 days postoperatively depending on the tumor extension according to the Hannover classification. There is a clear tendency that a higher Hannover grade leads to a higher probability of a persistent postoperative facial nerve dysfunction without reaching statistical significance (*p* = 0.980).

Out of the patients undergoing STR 8.9% were confronted with a new postoperative persistent facial nerve dysfunction still present 90 days postoperatively. In contrast, 2.3% of patients out of CR group have suffered facial nerve injuries (*p* = 0.172).

### Postoperative hearing

**Fig. 2:**
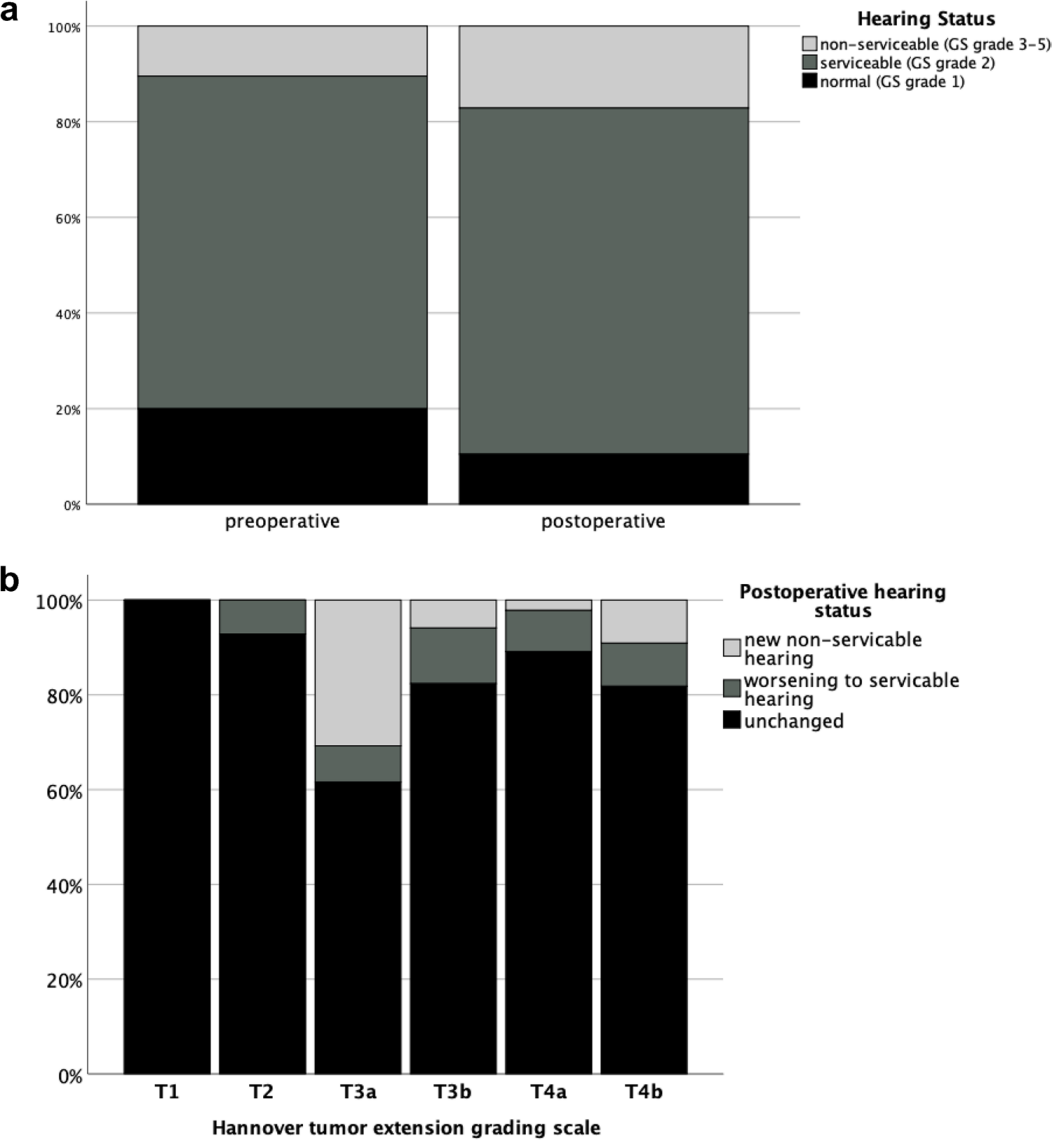
**a** Pre- and postoperative hearing status. GS, Gardner Robertson scale. **b** Postoperative hearing status in relation to the tumor extension according to the Hannover classification. There was no significant difference between hearing status and tumor extension grade (*p* = 0.126)

The majority of patients presented with impaired hearing prior to surgery (*n* = 71, 68.9%). Eleven patients had single-sided non-serviceable hearing (10.7%). The hearing was worse in 16 cases (15.5%) after surgery, 7 patients developed non-serviceable hearing (6.8%), and mild hearing impairment was observed in 9 patients (8.7%) (Fig. 2a). There was no difference between immediate, 30-day, and 90-day rates of new non-serviceable hearing. Figure 2b shows the hearing outcome depending on the tumor extension. Our data revealed a tendency for T3a tumors to be associated with a higher risk for postoperative worsening of hearing without reaching statistical significance (*p* = 0.126).

### Tumor recurrence

The overall recurrence rate was 8.7% (7/103). The median time to recurrence was 30 months (range: 18–50 months).

The recurrence rate depended significantly on the EOR (recurrence rate for CR: 0% (0/43), NTR: 10% (2/20), STR: 17.9% (7/39), *p* = 0.016).

Most patients were treated with GammaKnife when diagnosed with tumor recurrence (66.7%, 6/9). Three patients (33.3%) were surgically treated with two of them being treated with additional postoperative GammaKnife.

### Extent of resection

CR was performed in 41.7% (*n* = 43), whereas NTR was documented in 19.6% (*n* = 20) and STR in 38.2% (*n* = 39) (Table 1). In one case, there was no postoperative MRI available for defining the EOR. Of patients with STR, 33.3% received postoperative radiation therapy (GammaKnife: *n* = 10, 76.9%, conventional radiotherapy *n* = 2, 15.4%, and CyberKnife *n* = 1, 7.7%).

### Risk factors for primary outcome measures/adverse events

Several variables have proved to have a significant impact on more than just one quality indicator in vestibular schwannoma surgery. Overall, the degree of tumor extension, graded according to the Hannover classification scheme, has showed to significantly impact 30-day reoperation (*p* < 0.001), 30-day nosocomial infection (*p* = 0.015), postoperative hemorrhage (*p* < 0.001), and postoperative hearing loss (*p* = 0.026). In addition, hydrocephalus prior to surgery has shown to impinge upon different outcome values: 30-day reoperation (*p*-value < 0.001), 30-day nosocomial infection (*p*-value = 0.028), and postoperative hemorrhage (*p*-value < 0.001). Similarly, postoperative hydrocephalus (*p*-value < 0.001) and postoperative hemorrhage (*p*-value < 0.001) raise the likelihood of nosocomial infections.

A 30-day readmission was mainly affected by alcohol abuse (*p* = 0.019), depression (*p* < 0.037), and nicotine abuse (*p* < 0.037). Male patients were confronted with a significantly higher probability of a CFS leak (*p* = 0.033), whereas elderly patients faced a higher risk of postoperative hemorrhage (*p* = 0.006). Results from the univariate analysis are summarized in Table 3.

**Table 3:** Risk factors for the different outcome variables obtained in univariate analysis. All variables that are presented in Table 1 were subject to analysis. In this table only significant results (*p* < 0.05) are shown

30-day reoperation				
		Reoperation *n* (%)	No reoperation *n* (%)	*p*-value
Tumor size		Median, 2.90 (IQR, 2.1)	Median, 2.0 (IQR, 1.2)	0.022
	** < 2 cm**	6 (7.1%)	78 (92.9%)	0.015
	** ≥ 2 cm**	5 (26.3%)	14 (73.7%)	
Hannover classification	**1**	0 (0.0%)	2 (100%)	< 0.001
	**2**	0 (0.0%)	14 (100%)	
	**3a**	1 (7.7%)	12 (92.3%)	
	**3b**	3 (17.6%)	14 (82.4%)	
	**4a**	1 (2.2%)	45 (97.8%)	
	**4b**	6 (54.5%)	5 (45.5%)	
Hydrocephalus preop surgery	**yes**	5 (55.6%)	4 (44.4%)	< 0.001
	**no**	6 (6.4%)	88 (93.6%)	
30-day nosocomial infection				
		Nosocomial infection *n* (%)	No nosocomial infection *n* (%)	*p*-value
Hydrocephalus preop surgery	**yes**	2 (22.2%)	7 (77.8%)	0.028
	**no**	4 (4.3%)	90 (95.7%	
Hannover classification	**1**	1 (50%)	1 (50%)	0.015
	**2**	0 (0%)	14 (100%)	
	**3a**	0 (0%)	13 (100%)	
	**3b**	2 (11.8%)	15 (88.2%)	
	**4a**	1 (2.2%)	45 (97.8%)	
	**4b**	2 (18.2%)	9 (81.8%)	
30-day reoperation	**yes**	4 (36.4%)	7 (63.6%)	< 0.001
	**no**	2 (2.2%)	90 (97.8%)	
Hydrocephalus postoperative	**yes**	3 (33.3%)	6 (66.7%)	< 0.001
	**no**	3 (3.2%)	91 (96.8%)	
Postoperative hemorrhage	**yes**	3 (42.9%)	4 (57.1%)	< 0.001
	**no**	3 (3.1%)	93 (96.9%)	
30-day readmission				
		Readmission *n* (%)	Not readmission *n* (%)	*p*-value
Alcohol abuse	**yes**	2 (40.0%)	3 (60.0%)	0.019
	**no**	8 (8.2%)	90 (91.8%)	
Depression	**yes**	3 (27.3%)	8 (72.7%)	0.037
	**no**	7 (7.6%)	85 (92.4%)	
Nicotine abuse	**yes**	3 (27.43%)	8 (72.7%)	0.037
	**no**	7 (7.6%)	85 (92.4%)	
CFS leakage				
		CFS leakage *n* (%)	No CFS leakage *n* (%)	p-value
Sex	**Male**	8 (18.2%)	36 (81.8%)	0.033
	**Female**	3 (5.1%)	56 (94.9%)	
Postoperative hemorrhage				
		Hemorrhage *n* (%)	No hemorrhage *n* (%)	*p*-value
Hannover classification	**1**	0 (0%)	2 (100%)	< 0.001
	**2**	0 (0%)	14 (100%)	
	**3a**	0 (0%)	13 (100%)	
	**3b**	2 (11.8%)	15 (88.2%)	
	**4a**	0 (0%)	46 (100%)	
	**4b**	5 (45.5%)	6 (54.5%)	
Hydrocephalus preop surgery	**yes**	4 (44.4%)	5 (55.6%)	< 0.001
	**no**	3 (3.2%)	91 (96.8%)	
Age	Median (IQR)	70 (30)	52 (17)	0.006
Postoperative non-serviceable hearing				
		Non-serviceable hearing *n* (%)	No hearing loss *n* (%)	*p*-value
Hannover classification	**1**	0 (0%)	2 (100%)	0.026
	**2**	0 (0%)	13 (100%)	
	**3a**	4 (30.8%)	9 (69.2%)	
	**3b**	1 (7.1%)	13 (92.9%)	
	**4a**	1 (2.5%)	39 (97.5%)	
	**4b**	1 (10%)	9 (90%)	
Previous radiotherapy	**yes**	2 (50%)	2 (50%)	0.001
	**no**	5 (5.7%)	83 (94.3%)	

*IQR*, interquartile range

Multivariate analysis revealed a male sex as a predictor of CFS leak (OR = 4.15, 95%-CI = 1.03–16.68, *p* = 0.045). Moreover, age was found to be an independent risk factor of postoperative hemorrhage (OR, 1.12; CI, 1.02–1.24; *p* = 0.025). Additional potential risk predictors retrieved from univariate logistic regression analyses did not prove sufficient significance in the multivariate setting.

## Discussion

Despite advances in surgical techniques and perioperative management, short-term postoperative complications, and adverse events in VS surgery remain present.

Our study provides an overview of common postoperative complications and tumor inherent adverse events in VS surgery with regard to currently applied quality measures and provides data on the 30- and 90-day postoperative outcome. It has been widely discussed that health care providers apply different quality indicators to measure the quality of the delivered care and to justify reimbursement processes. Most of these quality indicators are closely connected to the occurrence of postoperative complications [44]. This justifies the analysis of predictors associated with adverse events, also allowing the development of strategies for risk adjustment between different health care providers.

Several quality indicators have been suggested for the field of neurosurgery, among them 30-day readmission, mortality, reoperation, nosocomial, and SSI rate [12, 13, 34, 43, 45–47, 50]. However, we are convinced that a more disease-specific approach when using quality indicators will be advantageous, especially in VS surgery. In the following, we discuss the various existing quality indicators, their time frame, and introduce new, more disease-specific outcome measures.

### Classical quality indicators

Commonly used quality indicators for several surgical fields are the 30-day reoperation and readmission rate. The usefulness and limitations of these indicators and the need for adequate risk adjustment have been widely discussed [12–14, 45, 46, 50].

Reported 30-day reoperation rates and readmission rates at the respective center corroborate the existing literature: Patients were readmitted to the hospital within 30 days in 9.7% and underwent a second surgery in 10.7% compared to documented incidence rates of 11.0–17.27% for unplanned readmissions and 5.93–8.0% for unplanned reoperations, respectively [3, 22, 35, 37].

For a better understanding of these factors, the analysis of the reasons for readmissions and reoperations is mandatory. We found that most reoperations were performed due to postoperative hemorrhage (*n* = 6, 54.5%), followed by CSF leak (*n* = 2, 18.2%). These data are in accordance with other studies [3]. The main reasons for readmission were CSF leak (*n* = 5, 50%) and the onset of a delayed new delayed facial palsy (*n* = 3, 30%).

Factors associated with a higher risk for 30-day reoperation were tumor size, higher grade in Hannover classification, and presence of a preoperative hydrocephalus; neither of them can be modified by the surgeon. On the contrary, univariate analysis revealed alcohol and nicotine abuse as well as depression being associated with readmission.

Thanks to an evolution in microsurgical techniques, 30-day mortality rates could have been drastically reduced from more than 10% to less than 1% over the past decades, which coincide with our cohort [3, 24, 33, 52]. The fortunately low incidence rate of perioperative mortality does not qualify this rate as an appropriate quality indicator for VS surgery due to lack of risk associated factors, impeding risk adjustment.

We found the nosocomial infection rate (5.8%) to be in line with established literature values ranging from 0.24 to 10% [3, 24]. Risk factors for nosocomial infection in our collective were pre- and postoperative hydrocephalus, higher Hannover tumor classification, early reoperation, and postoperative hemorrhage. All these factors are linked to prolonged length of hospital stay and intensive care unit (ICU) stay, predisposing for the development of nosocomial infections [43, 48], and again underlining the importance of adequate risk adjustment when using this variable as a quality indicator.

### CSF leak

CSF leak was implicated in half of all readmissions and 18.2% of all reoperations within 30 days and 40% of all reoperations within 90 days. Overall, an incidence of 10.7% within 90 days is in accordance with recent findings ranging from 6.0 to 14.1% [1, 2, 11]. With respect to the high impact of CSF leak on the aforementioned quality measures, integrating means to minimize the risk for CSF leak into clinical practice is highly advisable. While recent studies found obesity to be a prognostic factor of CSF leaks [21], our analysis could not confirm this relationship and revealed male sex (OR, 4.15) in accordance with a large series of 6820 patients to be at greater risk for CSF leaks [1].

### New onset of facial nerve palsy

A major challenge in VS surgery is the preservation of the facial nerve function as it inherently entails—despite constant enhancement in microsurgical techniques and monitoring modalities—a considerable risk of intraoperative damage of the facial nerve [40, 42].

Overall, 6.1% of all patients in our cohort developed a persistent facial nerve dysfunction (≥ HB III) still persistent at 90 days postoperatively—a relatively low value compared to reported incidence rates of facial palsy ranging from 4.8 to 41% [8, 16].

Based on the premise that both preservation of facial nerve and total resection of large tumors may be highly challenging, the concept of functional sparing surgery, condoning a near-complete tumor resection is preferred by the majority of VS surgeons, including our neurosurgical department [8, 31, 39]. Together with adequate intraoperative facial nerve monitoring, the rate of postoperative facial nerve palsy could be lowered considerably during the last decade [23, 54].

The rate of postoperative persistent facial nerve dysfunction (≥ HB III) can be seen as a useful quality indicator when assessing outcome in VS surgery. Obviously, this indicator has to be evaluated in relation to the EOR, aiming at maximal safe resection, while preserving the facial nerve integrity. Possible variables for preoperative risk adjustment and benchmarking purposes are tumor size [4, 20], history of previous VS surgery [18, 54], cystic tumor morphology [53], all factors that have been shown to be associated with a higher risk for nerve damage. A further risk factor is the degree of adhesiveness [4, 53]; however, this variable is not appropriate for risk adjustment as it cannot be clearly evaluated preoperatively.

In accordance with other studies [3, 4, 23, 36, 54], our data show that the EOR is not a predictive risk factor of postoperative facial nerve palsy, providing again evidence for maximal safe and facial function sparing resection.

### Postoperative non-serviceable hearing

An important factor that has a huge impact on the patient’s quality of life is postoperative hearing function [55]. Postoperative manifestation of single-sided non-serviceable hearing can be likewise seen as an indicator for quality. Tumor size and previous radiotherapy were identified as risk factors for hearing loss in our collective. In addition, impaired preoperative hearing has been found as a risk factor [5, 57]. These variables should be considered for preoperative risk adjustment. The rate of postoperative non-serviceable hearing was 6.8% in our cohort, compared to reported rates of 8.0%–47.0% [28, 32].

### Timing of measuring of quality

Most classical quality indicators refer to adverse events within a time period of 30 days after surgery (e.g., 30-day reoperation, readmission, mortality, and infection). We analyzed in addition the matching 90-day rates and could not show that the prolonged observation period detects other complications than CSF leak. In the respective cohort, 36.4% of all documented CSF leaks occurred more than 30 days after surgery. We therefore suggest to add “CSF leak” as a quality indicator covering the first 90 postoperative days, while keeping readmission, reoperation, mortality, and infection rates in the classical 30-day period.

In contrast, the rate of facial nerve dysfunction should not be evaluated before 3 months as an initial postoperative worsening of the facial function is likely to occur and recover, as both our data (30-day facial nerve dysfunction rate of 16.5% vs. 90-day rate of 6.1%) and many other studies could show [8, 54]. We did not document improving of hearing function during the 30- and 90-day postoperastive course, so that this outcome can be used as an immediate postoperative quality indicator.

**Fig. 3:**
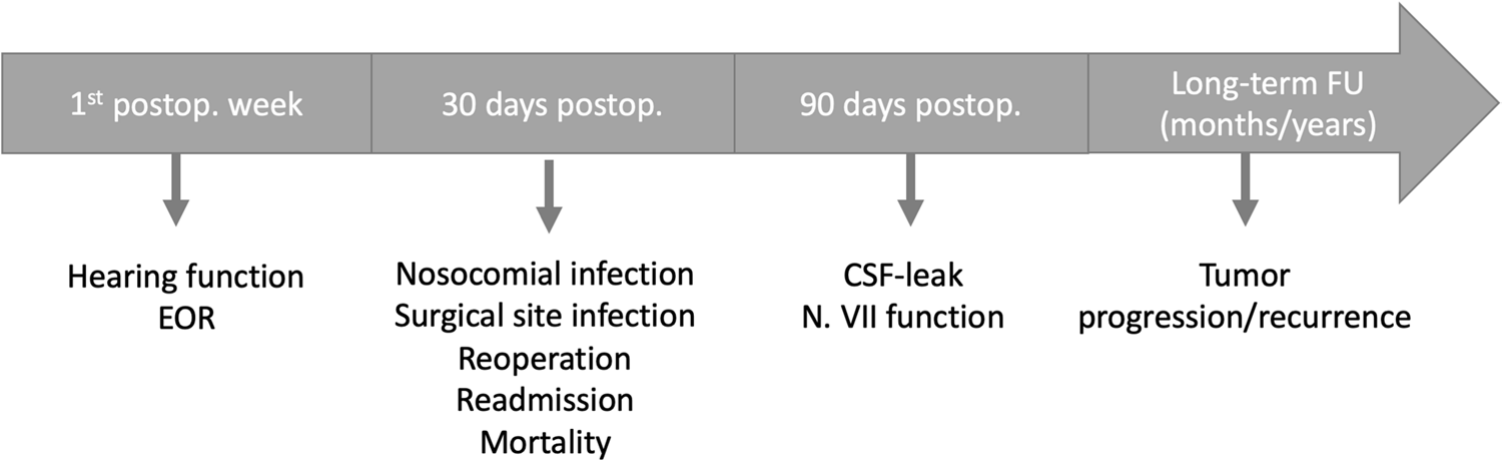
Recommendation for postoperative follow-up and recommended time for obtaining the various quality indicators

Tumor control and recurrence rate are subject to long time outcome and should be evaluated after months/years. We summarized the recommended time frames for all discussed quality indicators in Fig. 3.

### General aspects for assessment of quality and application of quality indicators in VS surgery

A common limitation of the suggested and already in some extent used quality indicators is their neglect of the long-term outcome, especially regarding tumor control. Extent of tumor resection has a strong influence on tumor recurrence [38, 49]. Therefore, outcome in VS surgery should always be evaluated on the background of maximal safe resection. If safe resection is not possible, combination of intended submaximal resection followed by radiosurgery showed promising results [51].

Tumor size appears to be a predominant factor when anticipating adverse events and outcome measures and therefore has to be considered as the main variable for risk adjustment when applying quality indicators.

A further quality indicator that could be suitable for vestibular schwannoma surgery—but that was not in the scope of our study—is quality of life (QoL) of patients. Surprisingly, the impact of hearing loss, facial nerve function and tinnitus on QoL is less by comparison to ongoing dizziness and headache [7].

### Limitations

The study is limited by the fact that data have been derived from a single hospital. However, all surgeries were carried out by the same surgeon. Despite these constant conditions assuring highly reliable results, their generalizability deserves additional discussion.

The data collection period extended from January 2013 and May 2019. During long observation periods, clinical procedures may have changed, and new surgical technologies could have been developed. However, all relevant international and national guidelines for the treatment of VS have not substantially changed. VS occurring with and incidence of 11 per million people, a comparatively long observation interval was inevitable for providing a robust volume of 103 cases [29].

Furthermore, the actual readmission rate could be higher than documented. Patients may have been readmitted to a different hospital and thus not accurately recorded in this study.

Multivariate analyses of the outcome variables “30-day reoperation” and “30-day readmission” have proved to be difficult. A larger sample size would have led to higher statistical power. However, univariate analyses remain valuable for identifying clinically relevant prognostic factors for all outcome variables.

As the study is designed in a retrospective setting, it is prone to the biases associated with this research method. It is well known that there are several surgical approaches for VS surgery, each bearing different risks for the presented outcome and quality measures. Our data applies only to tumor resection via the retrosigmoid approach.

## Conclusions

We presented an overview of tumor inherent postoperative complications in VS surgery in relation to currently available quality indicators for the field of neurosurgery. Measuring the quality of care for certain diseases, the application of specific quality indicators appears to be useful. Our analysis has revealed three outcomes that appear useful as quality indicators in VS surgery: the occurrence of a CSF leak within 90 days, new and still persistent facial nerve palsy 90 days postoperatively, and postoperative hearing impairment to non-serviceable hearing. All outcomes are relatively common and easy to monitor. In contrast to already established classical quality indicators, which refer to the first 30 postoperative days only, longer observation periods (90 days) are warranted when applying CSF leak and onset of a new persistent facials nerve palsy as quality measures. Prospective multicenter studies are needed to evaluate the utility of the proposed indicators and to design adequate methods for risk adjustment.

## Abbreviations

OR: Odds ratio
CI: Confidence interval
CSF: Cerebrospinal fluid
VS: Vestibular schwannoma
STR: Subtotal resection
CR: Complete resection
SSEP: Somatosensory evoked potential
EMG: Electromyography
MEP: Motor evoked potential
DNS: Direct nerve stimulation
AEP: Acoustic evoked potential
GS: Gardner Robertson scale
